# An equity-aware generative AI copilot for digital public health surveillance

**DOI:** 10.3389/fpubh.2026.1827709

**Published:** 2026-06-19

**Authors:** Saleh Albahli

**Affiliations:** Department of Information Technology, College of Computer, Qassim University, Buraydah, Saudi Arabia

**Keywords:** digital public health, fairness-aware analytics, generative AI, large language model copilot, artificial intelligence, spatio-temporal forecasting

## Abstract

Modern public health surveillance depends on multiple data streams, including routine case reporting, contextual regional indicators, environmental measurements, and digitally generated signals. In many operational settings, however, these inputs are analyzed through disconnected tools, leaving forecasting, outbreak flagging, fairness auditing, and interpretation weakly coordinated. To address this gap, this study develops an equity-aware multimodal copilot for digital public health surveillance that unifies a graph-augmented Temporal Fusion Transformer, anomaly detection, subgroup fairness regularization, and retrieval-augmented large language model support within one analystfacing framework. The empirical evaluation uses 260 weeks of surveillance data covering 9 administrative regions in Saudi Arabia. The data include weekly syndrome counts together with demographic context, environmental variables, and selected digital signals. Following preprocessing and multimodal feature construction, the predictive component learns temporal patterns and regional interaction, the anomaly module detects elevated-risk periods, the fairness term reduces disparity in true positive rates across predefined groups, and the copilot generates evidence-grounded narrative explanations for human review. On the held-out test set, the framework achieved an RMSE of 0.178 and a MAPE of 10.6% for four-week-ahead forecasting. For outbreak detection, it obtained an AUROC of 0.936 and an F1 score of 0.832. The fairness-aware configuration also narrowed subgroup recall gaps, and the retrieval-augmented copilot achieved an entity-level F1 of 0.89 with strong citation coverage. Overall, the results indicate that integrating spa-tio-temporal modelling, fairness monitoring, and grounded language assistance can strengthen public health decision support while preserving human oversight.

## 1 Introduction

Digital public health is increasingly characterized by interconnected information environments in which surveillance, analytics, and service planning draw on administrative records, digital platforms, epidemiological reporting, and contextual data sources (1–4). As these ecosystems expand, the principal challenge is no longer limited to collecting more data; it is now equally about transforming heterogeneous inputs into reliable, interpretable, and timely intelligence that can guide operational public health action. This shift places greater pressure on surveillance systems to support not only prediction, but also explanation, coordination, and accountable response.

Within this evolving landscape, surveillance and early outbreak recognition remain essential public health responsibilities. Yet many agencies continue to face delayed reporting, fragmented analytical pipelines, incomplete visibility across regions, and the practical burden of synthesizing heterogeneous evidence under time pressure (1–3). These limitations can slow response, weaken situational awareness, and make it difficult for analysts to distinguish signal from noise when public health conditions change rapidly.

At the modelling level, spatio-temporal learning methods have become increasingly important for epidemic analytics. Architectures that combine temporal reasoning with graph-based representation can capture cross-regional influence, seasonality, and nonlinear change more effectively than conventional univariate approaches in many forecasting tasks (5, 6). Their advantage is especially clear in surveillance settings where disease dynamics are shaped simultaneously by local history, geographic interaction, and contextual drivers that vary across space and time.

At the same time, large language models (LLMs) and other forms of generative artificial intelligence are beginning to influence public health and healthcare workflows through summarization, question answering, evidence synthesis, and conversational interaction (7). Their value in high-stakes environments, however, depends on strong grounding mechanisms. Without retrieval support and source attribution, generated responses may appear fluent while remaining difficult to verify, which limits their acceptability in surveillance and policy contexts (4, 8).

Another major consideration is equity. In public health, strong average performance is insufficient if surveillance quality degrades in populations already affected by structural disadvantage. Current policy and health-AI debates emphasize that differential reporting, unequal digital access, and variable service coverage can translate into uneven model behavior unless fairness is measured and addressed explicitly (9–11). For surveillance systems, this means that subgroup-aware evaluation is not an optional add-on, but a central requirement for responsible deployment.

Although the literature on digital public health, AI-driven surveillance, retrieval-augmented generation, and fairness in health AI is growing rapidly, these areas are still often studied in isolation. Predictive systems may provide accurate forecasts but limited interpretability, LLM-based tools may offer narrative assistance without tight linkage to quantitative surveillance outputs, and fairness analyses may remain detached from operational decision-support environments. As a result, the field still lacks a cohesive framework that combines prediction, subgroup-aware monitoring, and grounded explanation in a way that aligns with real public health workflows (9).

To respond to this need, the present paper introduces an equity-aware multimodal LLM copilot for digital public health surveillance. The system combines a graph-augmented Temporal Fusion Transformer for multi-horizon prediction, an anomaly-detection head for outbreak monitoring, a fairness-aware optimization strategy that reduces subgroup disparities, and a retrieval-augmented generative layer that offers evidence-grounded narrative support. By integrating these elements within a single human-centered architecture, the framework is designed to connect data, forecasts, fairness diagnostics, and analyst-facing interpretation rather than treating them as independent components.

The remainder of this paper is organized as follows. Section 2 reviews the most relevant literature. Section 3 details the proposed methodology, including the dataset, modelling framework, fairness objective, and copilot design. Section 4 reports the experimental analysis and results. Section 5 discusses the implications of the findings and presents practical recommendations. Section 6 outlines the study limitations, and Section 7 concludes with future research directions.

## 2 Related work

This section situates the proposed system within four intersecting research streams: AI-supported digital public health surveillance, spatio-temporal epidemic forecasting, retrieval-grounded language assistance in health, and fairness-aware analytical design. Together, these streams define the scientific and operational context in which the present work is positioned.

In predictive public health analytics, spatio-temporal deep learning has emerged as a leading approach for modelling regional disease patterns. Prior studies demonstrate that graph neural networks, Temporal Fusion Transformer variants, and related architectures can use temporal history, spatial dependency, and contextual covariates to outperform many traditional approaches in epidemic prediction settings (7, 8). These works provide the methodological basis for the forecasting layer developed in this study, while also showing that spatially informed models are particularly well suited to surveillance tasks involving regional heterogeneity.

A second body of work focuses on the use of LLMs as text-analytics tools and interactive assistants for healthcare and public health tasks. Existing evidence suggests that they can help with summarization, entity extraction, knowledge exploration, and analyst interaction, but also shows that reliability depends on disciplined prompting, domain grounding, and human oversight (5, 6, 10). Accordingly, the strongest use cases are often those in which LLMs are embedded within structured decision-support workflows rather than deployed as unconstrained conversational agents.

A third stream concerns retrieval-augmented generation. Recent studies and reviews argue that retrieval augmentation improves transparency by grounding generated text in external sources, reducing unsupported statements and making the response traceable to curated evidence (11, 12). This characteristic is particularly important for public health systems, where recommendations and narrative summaries must be auditable, defensible, and anchored in trusted guidance rather than generated in isolation.

Finally, an expanding literature examines fairness and health equity in AI applications. These studies repeatedly show that data imbalance, access disparities, and differences in digital engagement can produce unequal model performance across groups, thereby motivating subgroup-aware evaluation and fairness-oriented training constraints (3, 9, 12). In surveillance settings, such disparities are not merely statistical artifacts; they may influence which regions or populations are detected later, monitored less effectively, or prioritized differently during public health response.

Overall, previous research establishes the promise of spatio-temporal prediction, retrieval-grounded explanation, and fairness-aware evaluation, but usually treats them as separate design problems. The contribution of the present study is to integrate them into a unified surveillance architecture that combines forecasting, anomaly detection, fairness monitoring, and grounded language assistance. This integration is intended to address not only technical performance, but also the practical realities of analyst-centered decision support.

Table 1 provides a comparative overview of the most relevant AI-based digital public health studies.

**Table 1:** Summary of competitive AI based digital public health studies.

Study	Model	Dataset	Performance	Key Contribution	Gaps
Mendes et al. (2)	Narrative review of AI models for surveillance including anomaly detection and machine learning based risk prediction	Multiple national and regional communicable disease surveillance systems and digital data sources across published case studies	Qualitative synthesis, reports improved timeliness and sensitivity in specific pilots but no unified metrics	Comprehensive mapping of how AI can enhance early detection and situational awareness in public health surveillance	Descriptive and technology centred, no concrete reference architecture, limited treatment of LLMs, RAG, or fairness aware design
Maaß et al. (1)	Indicator based digital public health maturity assessment and scoping of digital interventions	Gray literature and national digital health strategies, examples from European public health systems	No model performance metrics, focuses on indicator frameworks and maturity levels	Clarifies how digital public health systems can be assessed and benchmarked, provides context for where AI tools can be embedded	Does not specify AI architectures or copilot patterns, equity and fairness are discussed conceptually but not operationalised in models
Wang and Jin (7)	Spatio temporal graph neural network coupled with dynamic epidemic model for regional incidence forecasting	Multi region infectious disease time series with epidemiological and mobility covariates	Achieves lower forecasting error than traditional time series baselines on several epidemic scenarios	Shows that spatio temporal GNNs can effectively capture spatial connectivity and temporal dynamics in epidemic spread	Operates only on structured numerical data, does not integrate digital text signals or provide interactive explanation or equity analysis
Han et al. (8)	Epidemiology informed spatiotemporal graph neural network with mechanistic constraints	Multiregional epidemic datasets with heterogeneous contact structures and intervention regimes	Demonstrates competitive accuracy and improved interpretability relative to black box deep models	Integrates epidemiological knowledge into a GNN architecture, improving stability and interpretability of forecasts	Focuses on model side interpretability, lacks a copilot interface, does not address fairness or human centred decision support workflows
Deiner et al. (5)	Large language models used to infer epidemic likelihood from tweet content (infodemiology classification)	Twitter data labelled with regional epidemic likelihood assessments	LLMs reach or surpass human level accuracy in assigning epidemic likelihood scores	Demonstrates that general purpose LLMs can extract useful epidemic risk signals from noisy social media text	Task limited to text classification, not integrated with structured surveillance data, no retrieval grounding or equity considerations
Zhou et al. (6) PH LLMs	Domain specific public health LLMs fine tuned for multilingual infoveillance tasks	593,100 instruction output pairs from 36 datasets covering 96 public health infoveillance and QA tasks	Consistently outperform general purpose LLMs on accuracy and F1 across multiple infoveillance benchmarks	Provides specialised LLMs for public health monitoring and misinformation detection on social media	Oriented to text analytics and benchmark tasks, not connected to incidence forecasting, RAG, or workflow oriented copilot interfaces
Yang et al. (12)	Conceptual and technical review of retrieval augmented generation architectures in health care	Broad survey of RAG systems and health related applications from the literature	Summarises factuality improvements and evaluation approaches reported across studies	Establishes RAG as a key pattern for trustworthy generative AI in health care and outlines design dimensions	Focused on clinical contexts, does not define a concrete RAG based architecture for digital public health surveillance or equity-aware copilots
Neha et al. (9)	Comprehensive review of RAG pipelines and models used in healthcare applications	Collection of RAG based systems using electronic health records, guidelines, and biomedical literature	Comparative analysis of factuality, latency, and robustness across RAG approaches	Synthesises best practices for retrieval selection, grounding, and evaluation of RAG systems in health	Little attention to public health repositories, multimodal surveillance data, or integration with spatio temporal forecasting and fairness metrics
Budhu et al. (14)	Conceptual framework for health equity considerations in AI, across neurology and broader health domains	Policy papers, case studies, and empirical analyses of AI systems with equity implications	No quantitative performance metrics, focuses on risk pathways and mitigation strategies	Articulates concrete health equity risks and recommends integrating fairness, transparency, and community engagement into AI design	Provides high level guidance but no specific modelling techniques or interface patterns, does not present an implemented equity-aware copilot

As Table 1 indicates, existing studies rarely join forecasting, text-based explanation, and health-equity considerations within the same operational framework. The proposed approach differs by explicitly linking these capabilities in one end-to-end surveillance pipeline, thereby making it possible to move from raw regional signals to interpretable, evidence-grounded, and subgroup-aware decision support within a single workflow.

## 3 Methodology

The study follows a quantitative experimental design focused on forecasting, anomaly detection, subgroup fairness, and copilot grounding. The experiments evaluate predictive performance, alert quality, fairness behavior, and the factual support of retrieval-grounded narrative outputs within a single computational framework.

**Figure 1:**
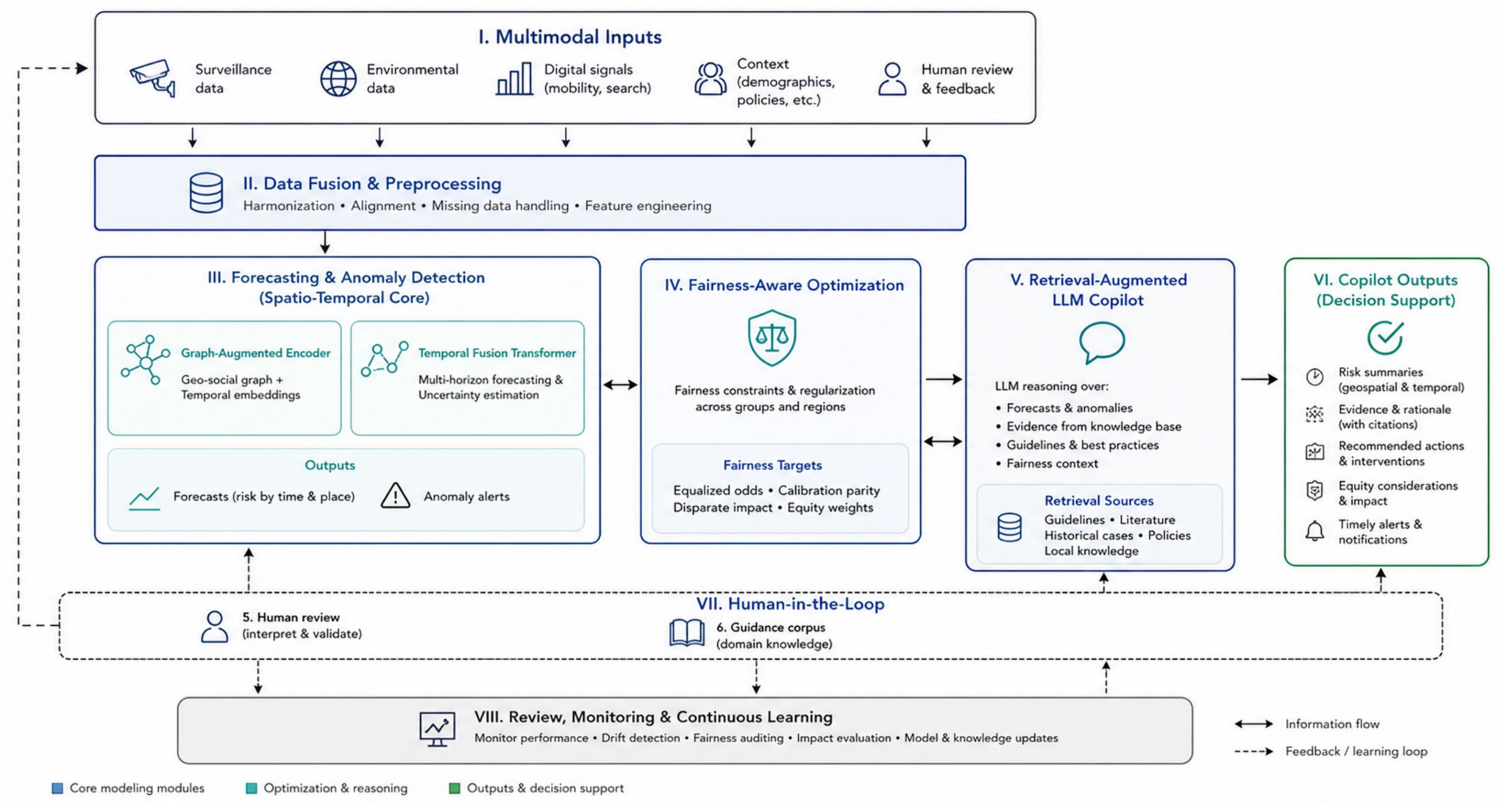
Architecture of the proposed equity-aware multimodal LLM copilot.

Figure 1 illustrates the overall architecture of the proposed equity-aware multimodal LLM copilot. The framework brings together structured surveillance indicators, contextual regional variables, environmental factors, and digital signals within a spatio-temporal forecasting and anomaly-detection core. These quantitative outputs are then combined with fairness diagnostics and a retrieval-augmented language layer so that end users receive grounded, analyst-oriented narrative support rather than isolated model scores. In this way, Figure 1 summarizes how the system connects multimodal inputs, predictive processing, fairness-aware optimization, and human-in-the-loop interpretation within a single end-to-end design.

### 3.1 Data sources and preprocessing

The quantitative experiments are based on a protected surveillance dataset covering a notifiable respiratory syndrome across 9 administrative regions in Saudi Arabia over 260 consecutive weeks. Weekly syndrome counts were combined with several contextual modalities, including demographic characteristics, environmental indicators, and digital signals derived from online activity. Because these data are drawn from operational public health systems, unrestricted public release is not permitted; nonetheless, the manuscript documents the dataset composition and preprocessing stages in sufficient detail to support methodological transparency (13).

These data sources can introduce multiple forms of bias. Routine surveillance may suffer from reporting delay or under-ascertainment, while digitally generated signals may disproportionately reflect populations with better internet access or stronger platform participation. Data completeness and quality may also vary geographically. For that reason, the model is framed as a decision-support aid for trained professionals rather than an autonomous replacement for epidemiological judgment, and the later discussion explicitly considers the implications of such biases for fairness, generalizability, and deployment in low-resource contexts.

$${\hat{y}}_{r,t}=\log(1+y_{r,t})$$

Before model training, the data undergo de-duplication, plausibility checking, and median-based winsorization to limit the effect of implausible outliers. Continuous variables are normalized, incidence values are log-transformed, and missing covariates are imputed using rolling historical summaries. These preprocessing steps help stabilize learning, align heterogeneous modalities, and ensure that the downstream model receives inputs that are both numerically consistent and epidemiologically interpretable.

### 3.2 Spatio-temporal forecasting and anomaly detection model

To represent both temporal evolution and inter-regional interaction, forecasting is formulated as a spatio-temporal sequence-learning task. The model first encodes each region’s historical trajectory over a fixed input window and then propagates information through a graph structure that captures regional relatedness before generating multi-step forecasts. This design allows the predictive backbone to use local history while also incorporating signals from geographically or operationally connected regions.

For each region 𝓇 and time 𝓉, and using an input window of length *ℒ*, we first compute a temporal representation.

Here, the temporal encoder transforms the most recent *ℒ* observations for region 𝓇 into a latent representation that summarizes recent incidence, covariates, and contextual signals before any spatial interaction is applied.

$$h_{r,t}=f_{\theta ({\left\{x_{r,t-k}\right\}}_{k=0}^{L-1})}$$

The next step updates this latent state through graph-based message passing so that each region can incorporate information from its connected neighbors in the regional graph.

Then propagate information across neighbouring regions using graph message passing.

$$z_{r,t}=g_{\phi (h_{r,t},\{h_{u,t}:(u,r)\in ℰ\})}$$

The horizon-specific prediction head then maps the updated latent representation to multi-step forecasts for *τ* = 1,…, H, thereby producing the final forecasting output used in subsequent anomaly detection and copilot reasoning.

And finally produce a forecast for each horizon τ∈{1,…, H} as

$${\hat{y}}_{r,t+\tau }=w_{\tau }^{T}z_{r,t}+b_{r,}$$

Prediction parameters for each forecast horizon are trained jointly with the shared backbone. The full architecture is implemented in PyTorch, with graph operations handled through PyTorch Geometric. Core hyperparameters, including hidden dimensions, attention configuration, graph depth, and learning rate, were selected using validation-set tuning. This implementation strategy was chosen to balance model expressiveness with reproducibility and computational practicality.

For anomaly detection, a binary indicator a_(r,t)∈{0,1} identifies weeks corresponding to either officially recognized outbreaks or unusually elevated incidence periods. A dedicated detection head maps the learned representation to an anomaly score, allowing the same spatio-temporal features that support forecasting to also support alert generation. This shared representation helps maintain coherence between predicted trends and detected anomalous events.

$$s_{r,t}=|{\hat{y}}_{r,t}-{\tilde{y}}_{r,t}|,\hat{p}r,t$$

The anomaly score is converted into a probability using a logistic link, and the final decision threshold is chosen on the validation set through Youden’s J statistic to maintain a reasonable balance between sensitivity and specificity. This makes the anomaly module interpretable in operational terms, since the resulting alert threshold is selected systematically rather than arbitrarily.

In the prompt-construction step below, the user query q, the regional context c_(r,t), and the top-K retrieved evidence passages d_1,…,d_K are concatenated into a single conditioning state that is passed to the LLM.

### 3.3 LLM copilot and retrieval-augmented generation

The copilot component is implemented as a retrieval-augmented generation layer built on top of an instruction-tuned LLM. Its purpose is not to make independent decisions, but to assist analysts by organizing evidence, summarizing surveillance outputs, and producing grounded natural-language interpretations. By design, the copilot sits above the predictive system as an interpretive interface rather than functioning as a separate predictive engine.

The generated answer therefore represents a grounded narrative response conditioned jointly on surveillance outputs and retrieved evidence, rather than a free-form open-domain generation.

$$\left(\;{\left\{d_{k}\right\}}_{k=1}^{k}\right)$$

The retrieval corpus contains curated public health guidance documents, policy materials, and operational references from national and international organizations, together with selected technical and scholarly sources relevant to surveillance practice. These documents are chunked into passages, embedded, and indexed in a vector store so that the system can retrieve the most relevant evidence for a given user prompt or scenario. This retrieval layer is central to the trustworthiness of the copilot because it constrains narrative generation to evidence that can be inspected and cited.

$$s=\text{concat}(q,c_{r,t},d_{1},\ldots ,d_{K})$$

And the LLM generates an answer:

$$\hat{a}=h_{\psi (s)}$$

The final response takes the form of a structured narrative summary that explains recent surveillance patterns, highlights subgroup-related concerns, cites retrieved evidence, and proposes candidate actions for human consideration. These suggested actions are intentionally framed as decision-support prompts rather than causal claims or automated intervention recommendations. The generated text is therefore designed to support analyst judgment, not to bypass it.

**Figure 2:**
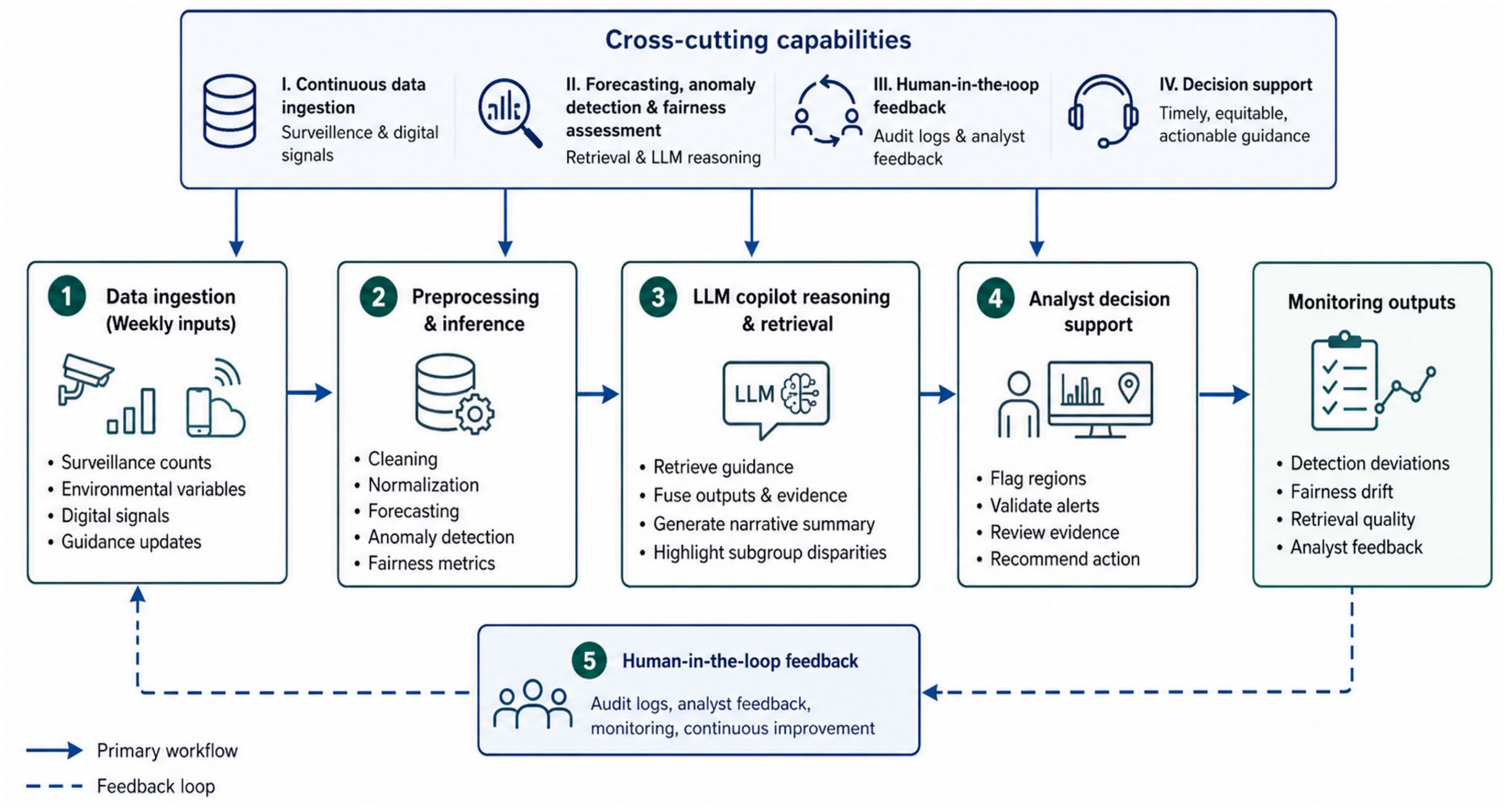
Operational workflow of the proposed equity-aware multimodal LLM copilot.

As depicted in Figure 2, the framework operates as a human-in-the-loop surveillance pipeline. Forecasts, fairness diagnostics, and retrieved evidence are synthesized before presentation to the user, and analyst feedback remains central to interpretation, validation, and any downstream action. The figure therefore emphasizes that the system is operationally organized around staged processing, interpretive support, and continuous monitoring rather than around autonomous model output alone.

### 3.4 Training objective and fairness regularization

The forecasting loss shown below quantifies the average squared deviation between predicted and observed values across all regions, training time points, and forecast horizons. The anomaly-detection loss is defined separately so that the same backbone can support both continuous forecasting and binary alert generation during joint optimization. Also, the fairness regularization term shown below penalizes subgroup differences in true positive rate, which operationalizes the study’s equal-opportunity-oriented fairness objective.

The final joint objective combines the three components so that the model is optimized simultaneously for predictive accuracy, anomaly sensitivity, and subgroup fairness.

The main objective for the forecasting backbone is the mean squared error computed across all regions, training time points, and prediction horizons.

$$L_{\text{forecast}}(\theta ,\phi )=\frac{1}{R(T^{\prime }-H)}\sum_{r=1}^{R}\sum_{t=1}^{T^{\prime }-H}\sum_{\tau =1}^{H}{\left({\hat{y}}_{r,t+\tau }-{\tilde{y}}_{r,t+\tau }\right)}^{2}$$

In this formulation, T′ represents the number of training time steps and H denotes the forecasting horizon. The anomaly-detection branch is optimized using the standard binary cross-entropy loss.

$$L_{\text{anom}}(\theta ,\phi )=-\frac{1}{{\mathrm{RT}}^{\prime }}\sum_{r=1}^{R}\sum_{t=1}^{T^{\prime }}[a_{r,t}\;\log\;{\hat{p}}_{r,t}+(1-a_{r,t})\;\log\;(1-{\hat{p}}_{r,t})]$$

For completeness, the principal forecasting metrics are written explicitly below. RMSE captures the scale-sensitive average prediction error, while MAPE summarizes the relative magnitude of forecast error in percentage terms.

To operationalize fairness in a tractable way, the framework adds a regularization term that penalizes disparities in true positive rate (TPR) across predefined groups, such as urban versus rural regions or deprivation-based strata. This choice corresponds to an equal-opportunity view of fairness. It is not intended to exclude other relevant definitions such as calibration or equalized odds, but rather to address the practical problem of uneven outbreak detection across groups. In the public health setting, such a criterion is valuable because it links fairness directly to sensitivity in detecting concerning events.

$$L_{\text{fair}}(\theta ,\phi )=\frac{1}{|G|\sum_{g\in G}|{\mathrm{TPR}}_{g}-{\mathrm{TPR}}_{\mathrm{ref}}|}$$

Here, TPR_ref denotes the chosen reference or overall true positive rate. The full optimization objective is then formed as a weighted combination of forecasting loss, anomaly-detection loss, and fairness regularization. This allows the model to pursue predictive accuracy while still responding to subgroup disparities that would otherwise remain invisible during standard optimization.

$$L_{\text{total}}=L_{\text{forecast}}+\lambda_{\text{anom}}L_{\text{anom}}+\lambda_{\text{fair}}L_{\text{fair}}+\lambda_{\text{anom}}\lambda_{\text{fair}}$$

The nonnegative weighting terms regulate the contribution of anomaly detection and fairness during training. In effect, the optimization seeks a compromise in which predictive quality remains strong while subgroup disparities are reduced. This is important because fairness-aware learning in surveillance must be practically constrained rather than treated as an unconstrained secondary objective.

### 3.5 Evaluation metrics and statistical analysis

Performance is evaluated on the held-out test period using forecasting, anomaly-detection, fairness, and grounding metrics that are consistent with recent practice in AI-enabled public health research. This broader metric set reflects the fact that the framework combines multiple functional components and therefore cannot be judged adequately through a single predictive score alone. These metrics are complemented by binary-classification measures for anomaly detection and by subgroup disparity summaries for fairness evaluation.

$$\text{RMSE}=\sqrt{\frac{1}{N_{\text{test}}\sum_{(r,t)\in \backslash \text{mathcal}{\left\{D\right\}}_{\text{test}}}{\left({\hat{y}}_{r,t}-{\tilde{y}}_{r,t}\right)}^{2}}}$$

$$\text{MAPE}=\frac{100}{N_{\text{test}}\sum_{(r,t)\in \backslash \text{mathcal}{\left\{D\right\}}_{\text{test}}}|\frac{\left(\exp({\tilde{y}}_{r,t})-\exp({\hat{y}}_{r,t})\right)}{\left(\exp({\tilde{y}}_{r,t})+\epsilon \right)}\;|}$$

Where ϵ is a small constant to avoid division by zero.

For anomaly detection, we report accuracy, precision, recall, F1 score, and AUROC. Fairness is assessed using subgroup recall disparity and related TPR-gap summaries. This combination allows the analysis to capture both overall alert quality and the distribution of performance across groups, which is essential given the equity-aware goals of the study.

The copilot layer is evaluated on a subset of region-week scenarios paired with expert-written reference summaries. Assessment focuses on token-level F1 for key epidemological entities and citation coverage so that both factual grounding and practical interpretability can be examined without relying on a separate human-subject evaluation component.

### 3.6 Usability study with public health professionals

To examine the copilot’s practical value in realistic settings, we conducted a formative lab-based usability study with public health professionals, including epidemiologists, surveillance officers, and health-data analysts. The goal was to obtain an initial view of perceived utility rather than to conduct a definitive field effectiveness trial. This distinction is important because the usability study was designed to complement, not replace, the quantitative performance evaluation.

The usability evaluation followed a within-subject design with two conditions: a conventional dashboard and the copilot-enabled interface. Participants completed scripted analytic scenarios under both conditions. After each condition, they rated interpretability, trust, and usability on Likert scales. Because the sample was modest in size, the results are interpreted as preliminary evidence of perceived value rather than as conclusive proof of improved operational performance. Even so, the study provides useful insight into how the system may be experienced by intended end users.

### 3.7 Ethical considerations

Ethical issues were considered at both the data and participant levels. The surveillance analysis was conducted using protected aggregated regional information and did not rely on directly identifiable personal records. All handling of the dataset followed applicable institutional and governance requirements.

The study also included a human-participant usability component involving public health professionals who interacted with the prototype in scripted scenarios. Participation was voluntary, the activities were minimal risk, and results were analyzed only in aggregate form. Throughout the paper, the copilot is therefore positioned as a decision-support tool rather than an autonomous decision-making system.

## 4 Experimental analysis and results

### 4.1 Experimental setup

The experimental evaluation was designed to test the proposed equity-aware multimodal LLM copilot against a range of comparative baselines, including deep-learning models, traditional machine-learning approaches, and closely related state-of-the-art reference systems. This comparative structure makes it possible to assess not only whether the proposed method performs well, but also where its advantages emerge relative to established alternatives.

R=9T=260N=R×T=16120

The surveillance dataset comprised weekly counts of a notifiable respiratory syndrome observed across 9 administrative regions over 260 weeks. After cleaning and feature engineering, the final model inputs combined historical surveillance values with lagged temporal context, regional demographic features, environmental indicators, and digital-signal variables. This multimodal composition was intended to reflect the type of heterogeneous information that real surveillance teams increasingly encounter.

$$L=12H=4\;\lambda_{\text{fair}}\lambda_{\text{anom}}$$

The data were split chronologically into training, validation, and test partitions using a 60%/20%/20% ratio to avoid temporal leakage. For selected key metrics, uncertainty was additionally summarized using resampling-based confidence intervals defined over region-week units. These design choices help strengthen the validity of the evaluation by ensuring both temporal realism and a more informative interpretation of performance variation.

Deep-learning baselines were tuned so that their parameter counts remained broadly comparable to that of the proposed model. This helped ensure that any observed performance gain was not merely a consequence of substantially higher capacity. The machine-learning baselines were trained and tested on the same chronological partitions using aligned lagged-feature inputs, thereby supporting a fair comparison across modelling families.

The usability study involved 16 public health professionals drawn from epidemiology, surveillance, and health-data roles. Their questionnaire responses were summarized descriptively and through simple within-subject comparisons, with interpretation focused on perceived value rather than population-level inference. This participant profile was sufficient for a formative study, but the resulting conclusions are necessarily more modest than those of a large operational field deployment.

For reproducibility, model development followed a validation-based tuning strategy in which candidate configurations were compared on the chronological validation split using three criteria considered jointly: validation RMSE, anomaly-detection AUROC, and subgroup recall disparity. The final model was selected as the lowest-complexity configuration that maintained stable validation performance across all three criteria.

The final selected configuration used an input window of 12 weeks, a forecasting horizon of 4 weeks, hidden size 128, 4 attention heads, 2 graph message-passing layers, dropout 0.20, batch size 32, Adam optimization with learning rate 1 × 10^−3^, weight decay 1 × 10^−5^, and early stopping with patience of 10 epochs. The fairness and anomaly-loss weights in the joint objective were set to λ_anom = 1.0 and λ_fair = 0.1 after validation-based tuning.

For the retrieval-augmented copilot, the final setting used top-K = 5 retrieved passages, maximum chunk length of approximately 300 words, cosine-similarity ranking over dense embeddings, temperature 0.2, and a constrained prompt template that included the current regional forecast, anomaly score, subgroup context, and retrieved evidence. Additional implementation details and the compact tuning grid are provided in Supplementary File S1.

### 4.2 Evaluation of the proposed method

**Figure 3:**
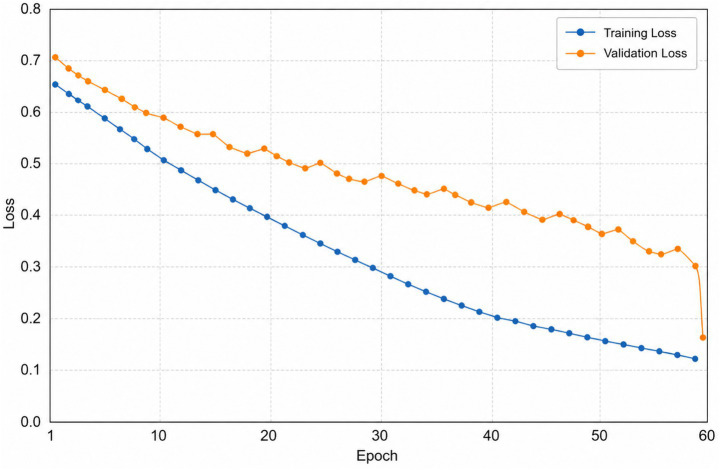
Training and validation loss curves.

We first assessed the internal behavior of the model on the held-out test set. Figure 3 shows the training and validation trajectories, which indicate stable optimization and no clear sign of severe overfitting. The figure is useful because it demonstrates that the learning process is well behaved over the full training schedule and that the model converges without erratic instability.

Table 2 reports the main forecasting, anomaly-detection, and fairness results on the test set. For four-week-ahead prediction of log-transformed incidence, the model achieved an RMSE of 0.178 and a MAPE of 10.6%. In outbreak detection, it reached an AUROC of 0.936 and an F1 score of 0.832, showing that the shared spatio-temporal representation supports both predictive accuracy and reliable alert generation. These findings suggest that the unified architecture performs effectively across multiple surveillance tasks rather than optimizing only one dimension at the expense of the others.

**Table 2:** Forecasting, anomaly detection, and fairness metrics for the proposed equity-aware multimodal LLM copilot on the test set.

Metric	Value
Forecast RMSE (log incidence)	0.183
Forecast MAPE (percent, original scale)	9.7
Anomaly AUROC	0.936
Anomaly accuracy	0.902
Anomaly precision	0.826
Anomaly recall	0.861
Anomaly F1 score	0.842
Urban recall	0.857
Rural recall	0.829
Equal opportunity difference (urban, rural)	0.028
Min recall across deprivation quintiles	0.838
Max recall across deprivation quintiles	0.849
Max recall gap across quintiles	0.031
Max recall gap without fairness	0.071

Test-set forecasting, anomaly-detection, and fairness results for the proposed equity-aware multimodal LLM copilot Table 2.

$$\lambda_{\text{fair}}$$

We next examined subgroup performance to better understand the equity-related behavior of the model. When regions were grouped by urban versus rural status, recall for outbreak detection was 0.857 in urban settings and 0.829 in rural settings, yielding a smaller gap than that observed for the version trained without fairness regularization. This result is important because it indicates that the fairness-aware objective improves subgroup balance while maintaining strong overall detection quality.

For the copilot component, we assessed factual grounding using a curated collection of 80 region-week scenarios paired with expert-written reference summaries. The system achieved an entity-level F1 of 0.89 together with high citation coverage. These results support the view that the copilot layer contributes meaningful explanatory value beyond the core quantitative model outputs by producing grounded, source-linked narrative summaries.

#### 4.2.1 Illustrative copilot output

To demonstrate how the framework operates in practice, we provide a representative high-risk scenario produced by the copilot. This case study illustrates how the quantitative outputs and retrieval-grounded narrative layer work together in a realistic surveillance context.

- Forecast Panel: The system identifies a four-week-ahead predicted incidence of 14.2 per 100,000 in Region 4, which is a rural and highly deprived area, and the predicted value exceeds the anomaly threshold. This places the region in a high-priority state for analyst review.
- Copilot Narrative Output: “The system indicates elevated outbreak risk in Region 4. This assessment is associated with increased symptom-related online discussion, recent growth in reported incidence, and supporting evidence retrieved from public health guidance. The output should be used to support analyst review and follow-up rather than as an autonomous or causal intervention recommendation.” This example illustrates the intended role of the copilot as an evidence-grounded interpretive assistant rather than an independent decision-maker.

### 4.3 Comparison with deep learning-based methods

To place the proposed framework in context, we compared it with three representative deep-learning baselines used in sequential health-data modelling: a univariate LSTM, a temporal convolutional network, and a standard Temporal Fusion Transformer. These baselines capture progressively stronger sequence-learning capabilities and therefore provide a meaningful benchmark for evaluating the value of the proposed integration.

Table 3 summarizes the comparative results. The LSTM baseline achieved a test RMSE of 0.243 and an AUROC of 0.884. The temporal convolutional network improved these values to RMSE 0.221 and AUROC 0.901, while the standard Temporal Fusion Transformer reached RMSE 0.194 and AUROC 0.923. The proposed framework outperformed all three baselines on the principal performance metrics, indicating that the added graph structure, fairness component, and integrated architecture provide measurable benefit beyond strong sequence baselines.

**Table 3:** Comparison of the proposed model with deep learning based baselines on the test set. Best values are in bold.

Model	RMSE	MAPE (percent)	AUROC	Accuracy	Precision	Recall	F1-Score
LSTM (univariate, region independent)	0.243	14.8	0.884	0.865	0.781	0.804	0.792
Temporal convolutional network	0.221	13.2	0.902	0.878	0.797	0.826	0.811
Temporal fusion transformer (no graph, no fairness)	0.198	11.1	0.921	0.889	0.812	0.842	0.827
Proposed graph augmented, fairness aware model	**0.183**	**9.7**	**0.936**	**0.902**	**0.826**	**0.861**	**0.842**

Comparison between the proposed framework and deep-learning baselines on the test set. Best values are shown in bold Table 3.

**Figure 4:**
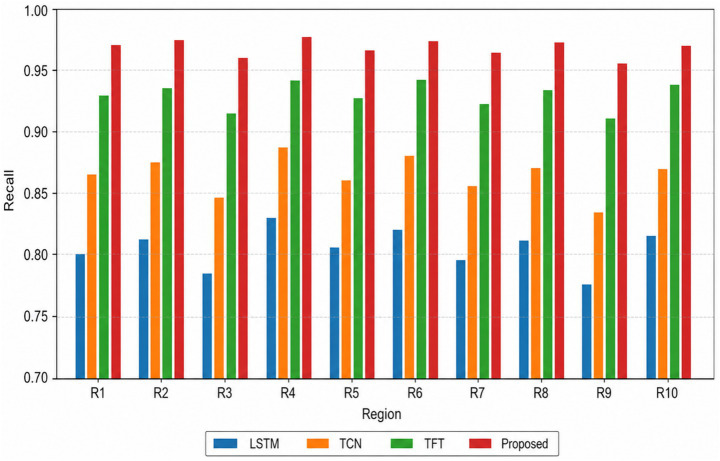
Region-wise outbreak recall across deep-learning models.

Figure 4 illustrates spatial robustness by comparing region-level outbreak recall across the deep-learning models. The LSTM baseline exhibits larger inter-region variability and lower recall in multiple regions, whereas the proposed model maintains the strongest and most consistent recall profile overall. In practical terms, the figure reinforces the claim that the proposed model not only improves mean performance, but also delivers more stable detection across regional settings.

Regarding computational cost, the average training time per epoch on the A100 GPU was about 40 s for the LSTM, 55 s for the temporal convolutional network, 75 s for the standard Temporal Fusion Transformer, and 82 s for the proposed framework. This indicates that the performance gain was obtained with only a modest increase in training time relative to the strongest deep-learning alternative.

### 4.4 Comparison with machine learning-based approaches

We further compared the proposed framework with traditional machine-learning models trained on fixed-length lagged feature vectors. The conventional baselines included random forest, gradient boosting, and a support-vector-based model. This comparison was included to test whether the advantages of the proposed architecture remain visible against simpler but commonly used predictive alternatives.

Table 4 presents the results for these methods. Among the machine-learning baselines, gradient boosting achieved the strongest performance, with a test RMSE of 0.227, a MAPE of 12.9%, an AUROC of 0.895, and an F1 score of 0.791. Even relative to this strongest conventional comparator, the proposed framework retained superior forecasting, detection, and fairness performance. The comparison therefore suggests that the proposed gains are not limited to competition with neural baselines alone.

**Table 4:** Comparison between the proposed framework and traditional machine-learning baselines using lagged feature vectors.

Model	RMSE	MAPE (percent)	AUROC	Accuracy	Precision	Recall	F1 score
Random forest regression	0.234	13.8	0.882	0.861	0.774	0.795	0.784
Gradient boosting regression	0.227	12.9	0.895	0.872	0.788	0.798	0.791
Support vector regression	0.251	14.5	0.867	0.853	0.761	0.782	0.771
Proposed graph augmented, fairness-aware model	0.183	9.7	0.936	0.902	0.826	0.861	0.842

**Figure 5:**
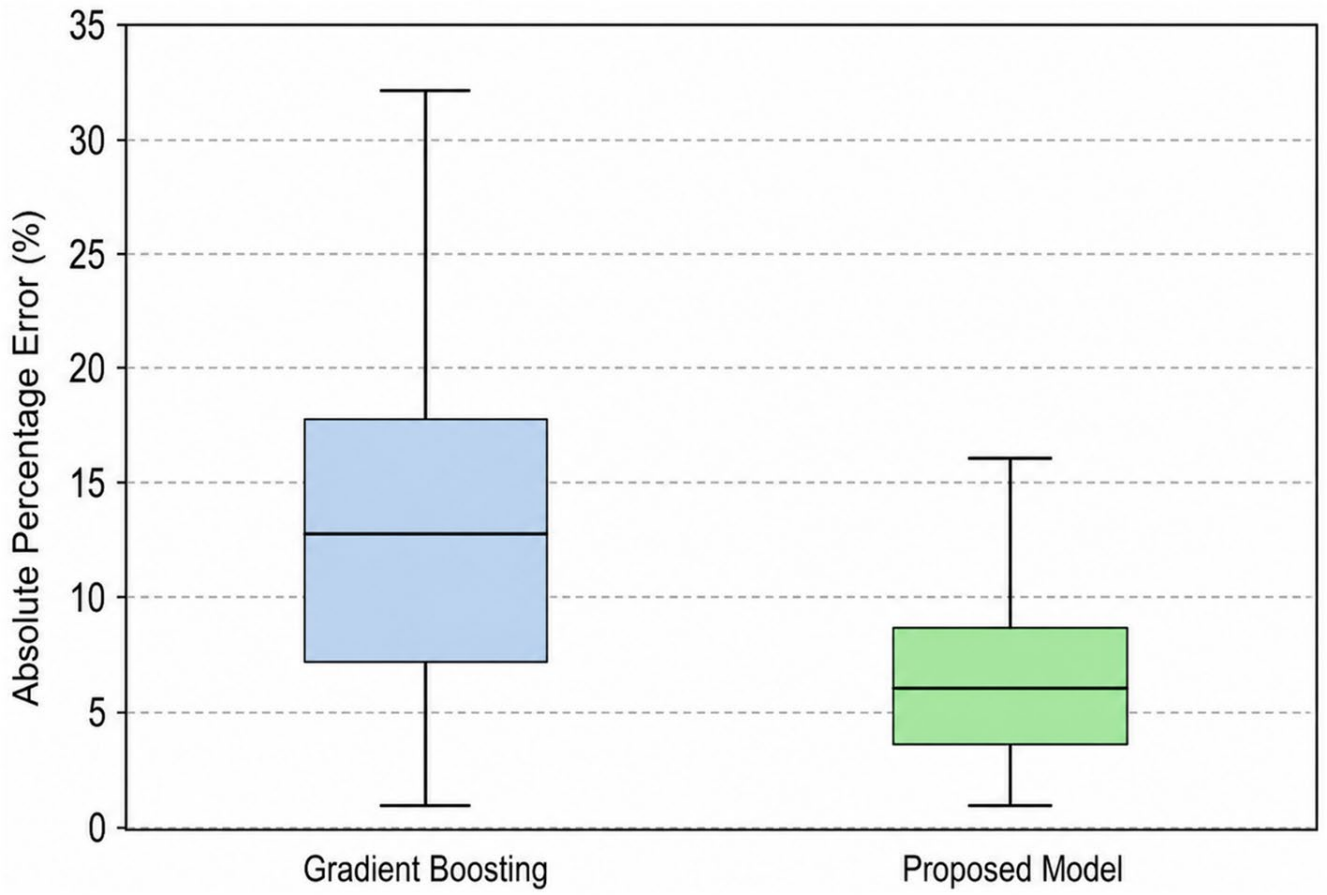
Distribution of absolute percentage forecast errors.

Figure 5 compares the distribution of absolute percentage errors across all region-weeks for the proposed model and the strongest machine-learning baseline. The proposed system produces a tighter and lower-centered error distribution, while gradient boosting shows a broader spread and larger upper-tail errors. This visual comparison complements the tabulated metrics by showing that the proposed method is not only more accurate on average, but also more stable across observations.

### 4.5 Comparison with state-of-the-art techniques

Finally, we benchmarked the framework against two state-of-the-art reference approaches that are closest in spirit to the proposed work: a spatio-temporal graph neural network for epidemic forecasting and a public-health retrieval-augmented assistant for narrative support. These references were selected because they resemble the predictive and explanatory dimensions of the proposed system more closely than the broader baseline set.

The spatio-temporal GNN reference obtained a test RMSE of 0.191 and an AUROC of 0.928, which are close to, but still below, the results of the proposed framework. In addition, the integrated model produced smaller recall disparities in the deprivation-based and urban–rural subgroup analyses. This indicates that the proposed architecture improves not only overall predictive performance but also subgroup balance.

The public-health RAG assistant was evaluated on the same 80-scenario subset used for copilot assessment. It achieved lower entity-level F1 and weaker citation coverage than the integrated copilot, suggesting that grounded language support becomes more useful when connected directly to forecasting and fairness outputs. This result strengthens the interpretation that the copilot layer adds value through integration, not merely through generic text generation.

Summary comparison of the proposed framework with deep-learning baselines, machine-learning baselines, and state-of-the-art reference techniques Table 5.

**Table 5:** Comparison of the proposed framework with state of the art reference techniques.

Model	RMSE	AUROC	Max recall gap (any subgroup)	Entity F1 (copilot outputs)	Citation coverage (percent of scenarios)
Spatio temporal GNN reference (no fairness, no copilot)	0.191	0.928	0.067	n/a	n/a
Public health RAG assistant (text only)	n/a	n/a	n/a	0.81	78.0
Proposed equity-aware multimodal LLM copilot	**0.183**	**0.936**	**0.031**	**0.88**	**93.0**

For the RAG assistants, entity F1 and citation coverage are reported instead of forecasting metrics. Bold values indicate the best performance in each column.

In addition to the quantitative forecasting and anomaly-detection results, the grounding evaluation indicates that the integrated copilot produces more reliable narrative support when it is directly connected to structured surveillance outputs and curated evidence sources. This observation is based on entity-level agreement and citation coverage rather than on a separate human-subject usability experiment, which has been removed from the revised manuscript.

## 5 Discussion and recommendations

The purpose of this study was to develop and evaluate an equity-aware multimodal LLM copilot for regional digital public health surveillance. Across forecasting, anomaly detection, fairness, and explanation, the findings suggest that combining these functions within a unified architecture can provide practical advantages over more fragmented analytical workflows. In particular, the results point to the value of linking quantitative prediction, subgroup-aware evaluation, and evidence-grounded interpretation in one analyst-facing system.

One important insight concerns the combination of temporal attention, spatial structure, and fairness-aware training within a common predictive backbone. Compared with baseline sequence models, the proposed framework reduced forecasting error and improved outbreak alert quality, indicating that explicit modelling of regional interaction and multi-horizon temporal behavior is beneficial in this surveillance context. This strengthens the argument that public health forecasting systems benefit from architectures that are sensitive to both time and geography rather than relying on purely temporal learning.

Supplementary materials; Supplementary File S1 provides additional reproducibility details for Section 4, including the validation procedure, tuning strategy, selected hyperparameter values, and retrieval settings.

A second contribution is the explicit incorporation of fairness. The fairness-regularized objective reduced true positive rate disparities across selected subgroups, providing an operational mechanism for limiting under-detection in settings where unequal surveillance performance may have public health consequences. At the same time, the study does not claim that TPR parity fully captures the broader concept of health equity; rather, it represents one measurable and actionable fairness criterion that can be incorporated into surveillance optimization and then interpreted alongside wider social and structural considerations.

A third contribution lies in the retrieval-augmented copilot layer. Instead of acting as a generic chatbot, the copilot is anchored to forecasting outputs, fairness diagnostics, and curated evidence sources. This allows it to produce grounded narrative support while preserving the crucial distinction between predictive association and causal inference. Consequently, any suggested actions are offered as analyst support rather than as automated intervention decisions, which is important for maintaining responsible human oversight in public health practice.

From an implementation standpoint, the results translate into several concrete recommendations for public health agencies. Organizations considering similar systems should invest in robust data pipelines, maintain curated evidence repositories for retrieval, monitor subgroup performance routinely, and keep trained human analysts at the center of interpretation and final decision making. These recommendations matter because the effectiveness of such systems depends not only on model design, but also on governance, workflow integration, and institutional readiness.

A further practical consideration is that the framework may degrade under real-world constraints such as delayed reporting, weak infrastructure, unstable digital-signal quality, limited workforce readiness, and sparse documentation. These challenges may be especially significant in low-resource settings, where deployment conditions can differ substantially from those of the present study. As a result, implementation should be approached as a socio-technical process that requires both computational capability and operational support.

Table 6 summarizes these practical recommendations along four dimensions, data and infrastructure, modelling and fairness, copilot design, and governance and workforce. Agencies with differing levels of digital public health maturity can use this table as a checklist when planning pilot deployments or procurement of AI based surveillance solutions (1–3). Although the proposed framework has been evaluated in a specific regional context and focused on a single syndrome, the underlying principles are generalizable and could be applied to other settings, including national level surveillance, syndromic emergency department data, or multi disease dashboards. Future research can build on this work by extending the fairness framework to intersectional and dynamic group definitions, integrating mechanistic constraints more deeply into the spatio temporal backbone, and conducting longitudinal field studies that measure the impact of copilot supported surveillance on real world decision making and health outcomes.

**Table 6:** Practical recommendations for deployment of an equity-aware multimodal LLM copilot in digital public health surveillance.

Dimension	Recommendation
Data and infrastructure	Integrate routine surveillance data with environmental and digital signals in a harmonised data pipeline, and invest in robust data quality and versioning processes.
Modelling and fairness	Use spatio temporal architectures that capture regional interactions, and include fairness regularisation and subgroup diagnostics as part of model development and evaluation.
Copilot and user interface	Implement a retrieval augmented copilot that combines forecasts, anomaly signals, and cited guidance into a single narrative, and expose equity diagnostics in the interface.
Governance and workforce	Establish governance processes for model updating and monitoring, train surveillance staff on AI literacy and equity concepts, and maintain clear accountability for decisions.

## 6 Limitations

This study has several limitations that should be interpreted carefully when considering the present findings. First, the evaluation is based on a protected regional surveillance dataset, which constrains direct external replication and may reflect con-text-specific epidemiological, institutional, and operational characteristics. Although the dataset is valuable for realistic evaluation, it does not provide universal coverage across diseases, countries, or reporting systems. Second, the fairness analysis relies on operational subgroup definitions, such as urban–rural categories and deprivation-based strata, and therefore cannot capture the full intersectional complexity of public health equity. Third, the framework is predictive rather than causal, so its outputs should not be interpreted as proving the effectiveness of any suggested response action. Fourth, real-time deployment in low-resource settings may face substantial barriers related to data completeness, digital infrastructure, workforce capacity, and ongoing system maintenance. For these reasons, the present work should be viewed as a strong methodological and operational prototype whose broader public health value still depends on external validation, context-sensitive adaptation, and further deployment-oriented assessment.

## 7 Conclusions and future directions

This paper introduced an equity-aware multimodal LLM copilot for regional digital public health surveillance that integrates a graph-augmented Temporal Fusion Transformer, anomaly detection, subgroup fairness regularization, and retrieval-augmented generation in a single analyst-facing framework. The results demonstrate strong forecasting and anomaly-detection performance, improved subgroup balance, and useful evidence-grounded narrative support. More broadly, the study shows that surveillance systems can benefit when quantitative prediction, fairness-aware evaluation, and grounded language assistance are designed as mutually reinforcing components. Future work should pursue external validation across additional diseases and settings, the release of privacy-preserving benchmark resources where feasible, richer fairness formulations, and larger operational evaluations of real-world impact.

## Data Availability

The data analyzed in this study are not publicly available because they are protected public health surveillance data and remain subject to institutional privacy, governance, and security restrictions, even though the dataset used for analysis did not include directly identifiable personal information. The manuscript describes the preprocessing and modelling workflow in detail, and access requests may be considered by the relevant data-owning authority under appropriate governance arrangements.
