# Supplementary material for: An equity-aware generative AI copilot for digital public health surveillance
Source: Front Public Health. 2026 Jun 19;14:1827709. doi: 10.3389/fpubh.2026.1827709 (PMC13328482; doi:10.3389/fpubh.2026.1827709)
Supplement: Supplementary file 1 [file Data_Sheet_1.PDF]

Supplementary File S1. Reproducibility Details for Experimental Analysis and Results

Associated manuscript: An Equity-Aware Generative AI Copilot for Digital Public Health Surveillance

S1. Purpose

This supplementary file reports additional quantitative implementation details requested for reproducibility. It summarizes the tuning strategy, validation procedure, final selected hyperparameter values, retrieval settings, and computational environment used in the experiments.

S2. Data Split and Validation Procedure

The full time series was partitioned chronologically into training, validation, and test segments using a 60%/20%/20% split. No random shuffling was applied at the time-series level in order to prevent temporal leakage. Model selection was based on validation performance rather than test-set tuning.

Candidate configurations were compared on the validation split using three criteria considered jointly: (i) forecasting RMSE, (ii) anomaly-detection AUROC, and (iii) subgroup recall disparity. The final configuration was selected as the model that achieved the best overall compromise across these criteria, rather than optimizing only a single score.

S3. Hyperparameter Tuning Strategy

A constrained validation-based tuning strategy was used. For the proposed spatio-temporal model, the search process varied the input window length, hidden dimensionality, number of attention heads, graph depth, dropout, and learning rate within practically motivated ranges. The final configuration is reported in Table S1.

Component	Search range / candidates	Final selected value
Input window length	{8, 12, 16} weeks	12 weeks
Forecast horizon	Fixed by study design	4 weeks
Hidden dimensionality	{64, 128, 256}	128
Attention heads	{2, 4, 8}	4
Graph message-passing layers	{1, 2, 3}	2
Dropout	{0.10, 0.20, 0.30}	0.20
Optimizer	{Adam, AdamW}	Adam
Learning rate	{1e-4, 5e-4, 1e-3}	1×10^-3
Weight decay	{0, 1e-5, 1e-4}	1×10^-5
Batch size	{16, 32, 64}	32
Maximum training epochs	Fixed upper bound	60

Early stopping patience	{5, 8, 10}	8 epochs
Gradient clipping	{0.5, 1.0, 2.0}	1.0
Fairness weight λ_fair	{0.05, 0.10, 0.20}	0.10
Anomaly weight λ_anom	{0.5, 1.0, 2.0}	1.00

Table S1. Validation-based tuning grid and final selected values for the proposed model.

S4. Retrieval-Augmented Copilot Configuration

The retrieval-augmented copilot was configured as a constrained analyst-support layer rather than an unrestricted open-domain chatbot. Documents in the retrieval corpus were chunked and indexed in a vector store. At inference time, the retriever returned a small set of top-ranked passages that were then combined with the regional surveillance context and user query before generation.

**Setting**	**Value**
Retrieval mode	Vector-based top-K retrieval over curated public-health and policy corpus
Top-K retrieved passages	5
Approximate chunk length	512 tokens
Chunk overlap	64 tokens
Generation temperature	0.20
Maximum generated response length	256 tokens
Output style	Structured surveillance summary with evidence-grounded recommendations for analyst review

Table S2. Final retrieval and generation settings for the copilot layer.

S5. Computational Environment

The experiments were implemented in PyTorch, with graph operations handled through PyTorch Geometric. Training was performed on an NVIDIA A100 GPU. In the final comparative analysis, the average epoch time was approximately 82 s for the proposed model, compared with about 40 s for the LSTM, 55 s for the temporal convolutional network, and 75 s for the standard Temporal Fusion Transformer.

S6. Notes on Reproducibility Scope

The surveillance dataset itself is protected and therefore not publicly released. For this reason, the present supplement aims to support high-level methodological reproducibility rather than full raw-data replication. The manuscript and this supplement jointly provide the model structure, data split logic, tuning strategy, selected hyperparameters, retrieval configuration, and computational setting required to understand and reproduce the experimental workflow on a comparable dataset.
